# Various hysterosalpingography findings of female genital tuberculosis: A case series

**Published:** 2013-06

**Authors:** Nargess Afzali, Firoozeh Ahmadi, Farnaz Akhbari

**Affiliations:** 1 *Department of Radiology, Faculty of Medicine, Islamic Azad University, Mashhad Branch, Mashhad, Iran.*; 2 *Department of Reproductive Imaging at Reproductive Biomedicine Research Center, Royan Institute for Reproductive Biomedicine, ACECR, Tehran, Iran.*

**Keywords:** *Genital tuberculosis*, *Infertility*, *Female*, *hysterosalpingography*

## Abstract

**Background: **Genital tuberculosis is a chorionic disease and mostly occurs by haematogenous spread from extra genital source like lungs, peritoneum, lymph nodes and bones. Transmission through a sexual intercourse is also possible. Since the majority of patients are in reproductive ages, involvement of fallopian tubes and endometrium cause infertility in patients.

**Cases:** Reviewing 4 cases of female genital tuberculosis, which referred to an infertility treatment center with various symptoms, we encountered various appearances on hysterosalpingography (HSG).

**Conclusion:** The genitourinary tract is the most common site of extra pulmonary TB. The primary focus of genital tuberculosis is fallopian tubes, which are almost always affected bilaterally but not symmetrically. Because of common involvement of fallopian tubes and endometrial cavity, disease causes infertility. Diagnosis is not easy because genital tuberculosis has a wide range of clinical and radiological manifestations with slow growing symptoms. Detailed hysterosalpingography finding may be helpful in better diagnosis of the disease. This case series aims to depict the various hystrosalpingographic appearances and pathology produced by tuberculosis and related literatures are reviewed in order to establish a better diagnostic evaluation of genital tuberculosis.

## Introduction

TUberculosis (TB) is regarded as the chronic disease with low grade symptomatology and few specific complaints in the world. Since the beginning of the 20^th^ century, the incidence of TB generally and genital TB specially has been steadily decreased in developed countries. But genital TB remains a major health problem in many developing countries, and is responsible for infertility in women HSG is one of the initial diagnostic procedures in the assessment of tubal and peritoneal factors leading to infertility (1-5). 

A review of the literature reveals that the highest incidence of TB is still in India (1). The real incidence of pelvic tuberculosis in Iran is still unknown. Several studies depicted TB is more common in patients at reproductive age (3, 6, 7). The actual incidence of genital TB cannot be determined accurately because there are no constant clinical signs and majority of them is discovered incidentally through infertility treatment. According to geographic location, socioeconomic and public health conditions, TB incidence is vary. There is direct relationship between prevalence of genital tuberculosis and pulmonary tuberculosis (1, 8-10). Probable symptoms with genital TB are infertility (most common clinical symptom), pelvic pain, abnormal vaginal bleeding, amenorrhea, vaginal discharge and post-menopausal bleeding (11-15). In some cases the disease presents with an abdominal mass or ascites and simulates an ovarian carcinoma. 

Peritoneal tuberculosis which is a form of abdominal tubercolosis, present in three forms: wet type with ascites, dry type with adhesions, and fibrotic type with omental thickening and loculated ascites (3). Sonographic features of wet tuberculosis (with ascites) include ascites, loculated fluid, thickened peritoneum, thickened omentum, endometrial involvement and adnexal masses (3, 4). Dry tuberculosis without ascites show endometrial involvements, adnexal masses, loculated fluid and adhesion (3). 

This case series depict the HSG appearances and pathology produced by tuberculosis. TB of the uterus is usually a silent infection with no apparent symptoms as the bacteria may remain latent in your system for as long as 10-20 years. However, some of the symptoms to watch out for include: menstrual disturbances, (sudden) weight loss, unexplained low or high grade fever over a prolonged period, pelvic pain, vaginal discharges, and infertility. 

Previous history of tuberculosis or a history of exposure to this disease should be considered. Histopathologies by endometrial biopsy and premenstrual or menstrual blood culture and demonstration of mycobacterium in the genital tract are useful tool for diagnosis. Abdominal or vaginal exam may be normal. Positive Monteux test and increased ESR (estimated sedimentation rate) in blood exam are nonspecific in diagnosis. HSG and pelvic sonography may be of great value in diagnosis. 

## Case report

**Case 1**

A 20 year old woman was referred to Royan institute for infertility treatment for secondary infertility. Her menstrual cycle was regular and her menarche was at 13 years. She got married 6 years ago and after one year had born a healthy baby via normal vaginal delivery but his child died because of an unknown etiology in 6 months. There was no positive history of TB in the family. Physical examination was normal except for trichomona infection in vaginal exam which was treated. 

**Figure 1:**
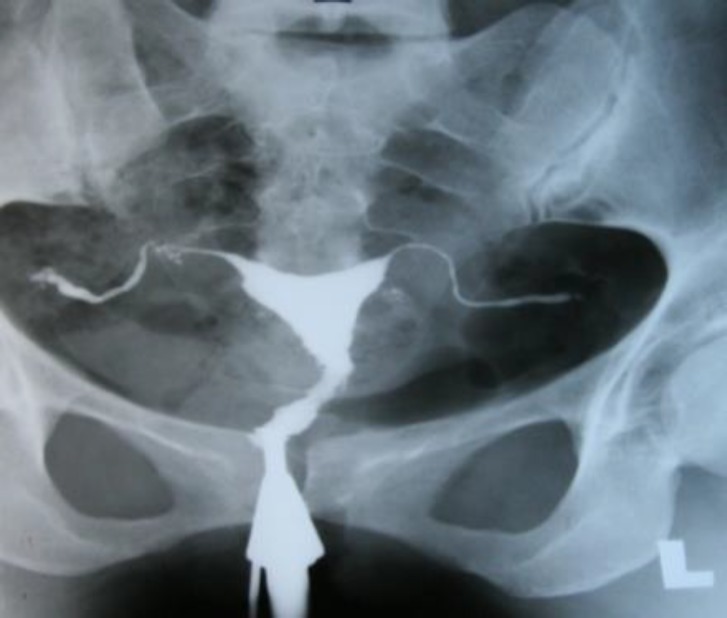
Case1: Uterine cavity with normal shape and size. Also there is rigid pipe stem appearance of the fallopian tubes

Mantoux test was positive as 30 millimeter diameter. HSG revealed endometrial cavity with normal shape and size. Interstitial and isthmus in both tubes were opacified but through ampullary region contrast medium were not passed that demonstrate bilateral fallopian tube obstruction. Rigid and fixed views shows pipe stem view in HSG (Figure1). In hysteroscopy endometrial cavity, cervical canal and vagina appeared normally. Laparoscopy revealed tubercles on peritoneal surface which was positive for TB in culture. She was treated by anti-tuberculosis drugs for 8 months. 

**Case 2**

A 25 year old woman with primary infertility who has regular menstruation and the menarche was at 12 years. Mantoux test was positive as 40 mm and chest X-ray revealed bilateral hilar calcifications. HSG showed normal cervix with irregular uterine cavity and clover leaf appearance which suppose uterine cavity adhesions. 

**Figure 2:**
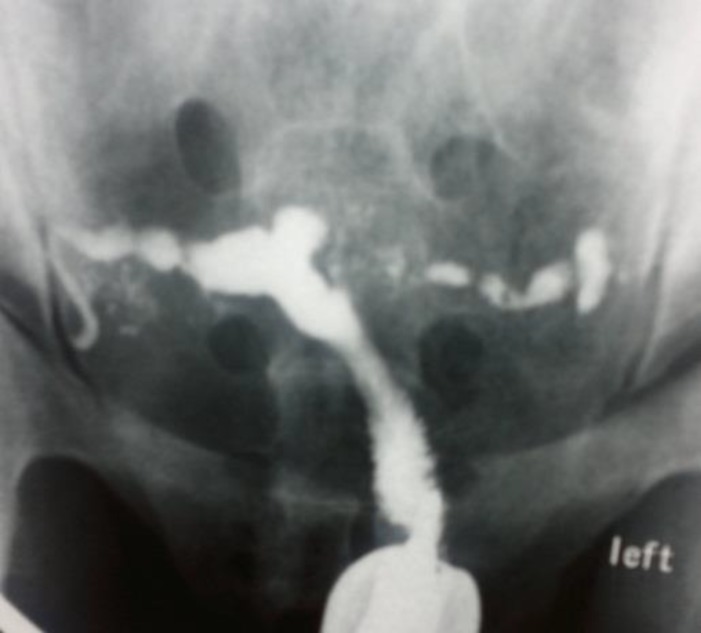
Case 2: Irregular uterine cavity and clover leaf appearance. Irregular border and beaded appearance in both fallopian tubes

Fallopian tubes had irregular border with beading appearance, contrast medium passage was not detected in pelvic cavity but venous extravasations was seen (Figure 2). Hysteroscopy findings show moderate to severe adhesions in uterine cavity. Menstrual blood smear was positive for acid fast bacillus and multidrug treatment was prescribed. 

**Case 3**

**Figure 3 (A, B, C):**
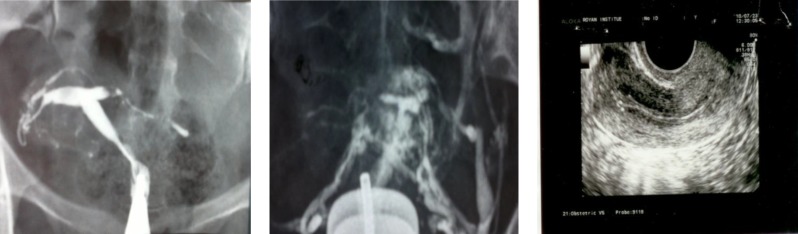
Case 3. A) HSG 4 years ago, bilateral tubal obstruction with golf-club appearance, B) Recent HSG revealed T shaped uterus, Venous and lymphatic intravasation. C) Uterine ultrasound, tiny foci of calcification within the endometrium which shows some degree of adhesion

A 33 years old women with primary infertility, who had the positive history of lung tuberculosis which was treated 12 years ago. The physical exam was normal and PPD was negative. HSG which was done 4 years ago showed small endometrial cavity and bilateral tubal obstruction in isthmus region and Golf club appearance (Figure 3A).Recent HSG (4 years later) revealed extent of involvement of uterine cavity with T-shape appearance. 

There were severe venous and lymphatic system extravasations so detecting fallopian tubes was impossible (Figure 3B). In uterine ultrasound there were tiny foci of calcification within an irregular endometrial which show endometrial adhesions that adjust HSG findings (Figure 3C). Hysteroscopic findings revealed severe adhesions which were resected as possible. There was no intact endomtrium so biopsy was not done and she was advised for surrogacy. 

**Case 4**

**Figure 4:**
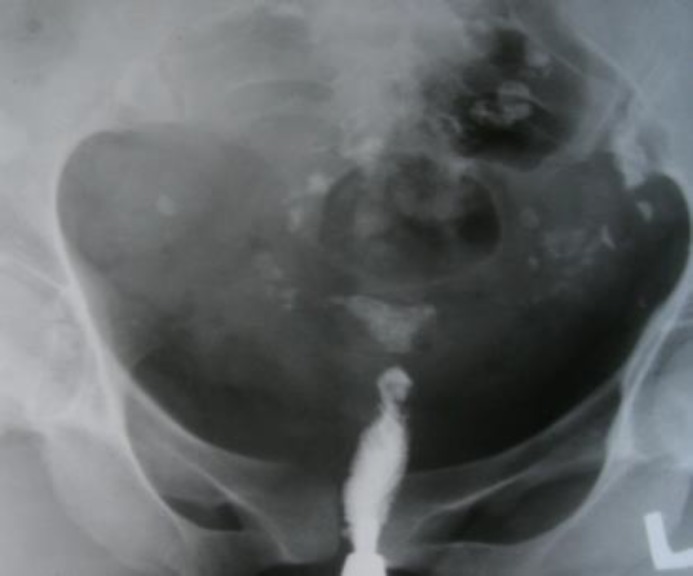
Case 4: Small uterine cavity and linear streaks of calcification in the course of the fallopian tube (Tubal calcification).

A 29 year old woman referred to a gynecologist with chronic pelvic pain, constipation and primary infertility. Menstrual cycle was irregular with hypomenorrhea in last three years. There was positive history of tuberculosis in patient's father which was living with her. Chest X ray was normal. HSG revealed multiple foci of calcification in pelvis and in the course of fallopian tubes (at plain film), small endometrial cavity and beaded appearance of tubes after instillation of contrast (Figure 4). Laparoscopy showed frozen pelvis, multiple fibrous bands in pelvic cavity and adhesion of peritoneum to the bowels. Endometrial biopsy cultured for acid fast bacillus was positive. The patient was treated with multidrug anti-tuberculosis drugs. Written consent was given from all 4 cases before study.

**Figure 5:**
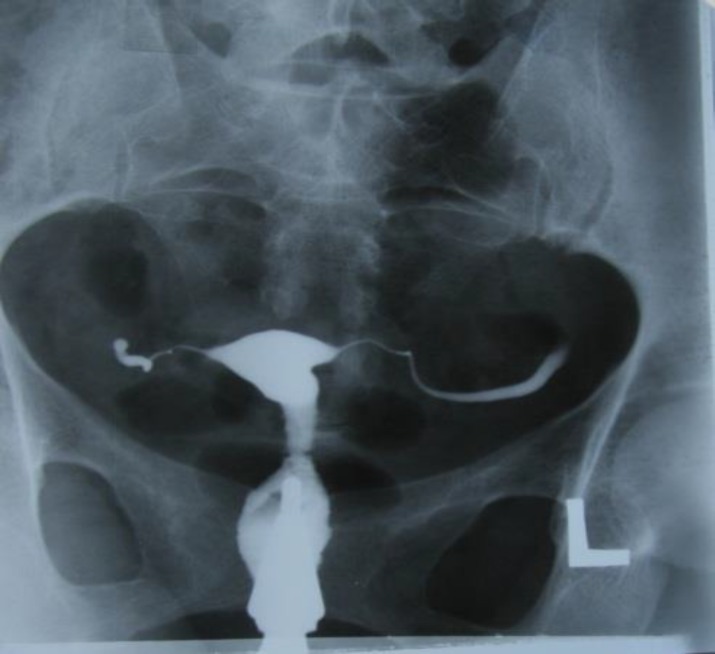
Occulsion in both distal isthmic region of fallopian tubes with pipe stem appearance

**Figure 6:**
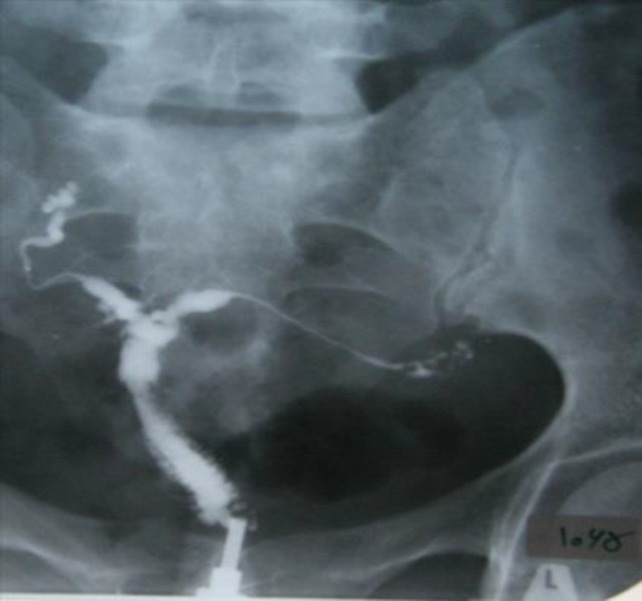
Small uterine cavity with irregular contour and resembling septate appearance. Diverticular outpouching around ampula in both fallopian tubes which make tuffed appearance

**Figure 7:**
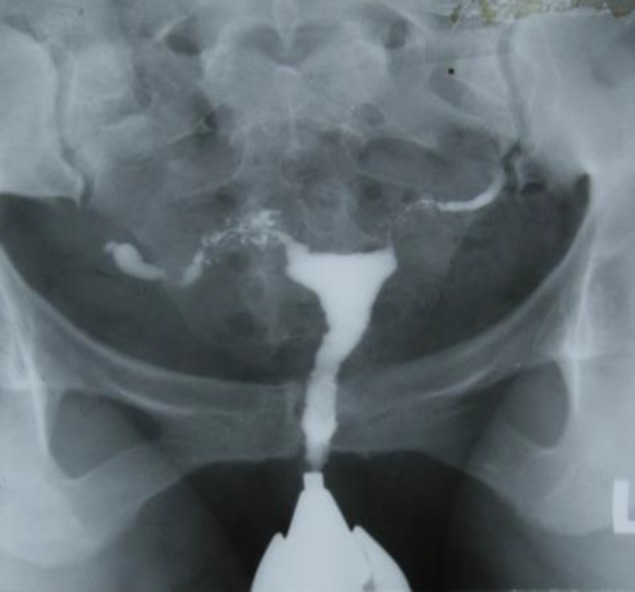
Normal shape of uterine cavity, irregular contour and diverticular outpouching which surround isthmus of right fallopian tube and make SIN appearance

**Figure 8:**
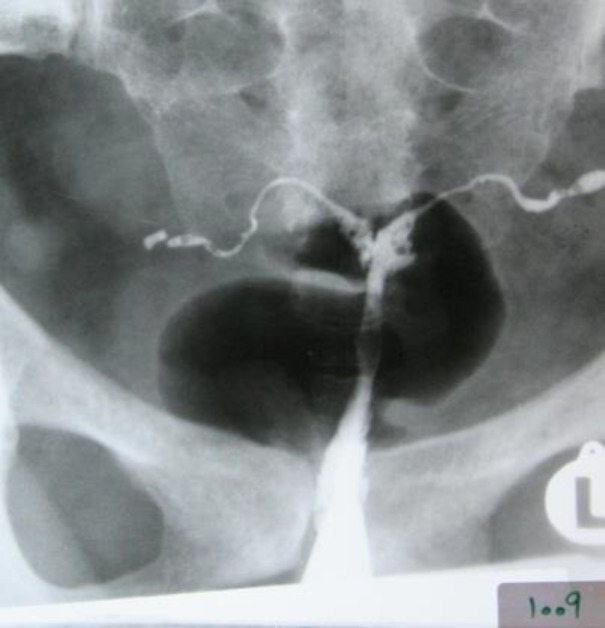
Small size of uterine cavity with irregular contour and septate appearance. Obstruction in distal isthmic portion of both tubes and golf club appearance

**Figure 9:**
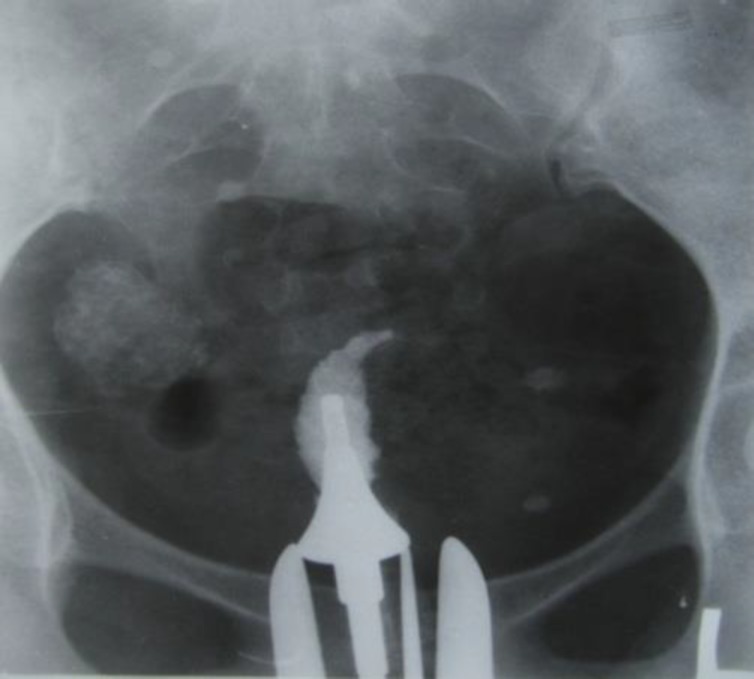
Complete obstruction of uterine cavity with glove’s finger appearance. Pelvic calcification (probably lymph node calcification) is detected

## Discussion

Tuberculosis (TB) remains the most common worldwide cause of mortality from infectious diseases (15). Genital tuberculosis with its variable presentation is a challenging problem for the gynecologist (11). About 20% of patients with genital TB give a family history of TB (as in case 3 and case 4). Hassoun *et al* in a 10-year study reported that 1.8% of all tuberculosis cases may have a genito-urinary site (1, 15). Abdominopelvic and peritoneal tuberculosis are not common. Peritoneal tuberculosis and tubo-ovarian lesions have usually minimal findings at CT and frequently misdiagnosed with peritoneal carcinomatosis. MRI is useful for the diagnosis of tubo-ovarian lesions. These lesions presented as no dularities along tubo-ovarian surface. 

However they are nonspecific and could be seen in peritoneal seeding by malignant tumors. Although regular pattern of small nodularities along the peritoneum at MRI are helpful findings which are proposed tuberculous peritonitis (14, 16). About 75% of cases with active genital tuberculosis have a normal chest X-ray, so a normal chest X-ray cannot exclude the diagnosis of genital tuberculosis (For instance case4 had normal chest X-ray but had infertility due to genital TB) (10). Tubal tuberculosis spreads to the endometrium in approximately one half of the cases; therefore a negative culture from uterine curetting does not exclude the diagnosis of genital tuberculosis (4). 

The fallopian tube is affected in almost all patients with active genital tuberculosis (15, 17, 18). The endometrium is involved in 50 % of all patients, the ovary in 20% of cases and the cervix in 5% of patients (17). In spite of significant technological advances in imaging noted with the advent of ultrasonography, CT and MRI; hystrosalpingography remains the gold standard in evaluating the internal architecture of the female genital tract and fallopian tubes. Also it is the most helpful procedure in suggesting the diagnosis of genital tract TB in patients investigated for infertility. 

Tubal lesions in tuberculosis have various appearances in HSG. These features vary from non-specific changes such as hydrosalpinx and strictures to specific patterns such as "beaded tube", (case 2) "golf club tube", (case 3) "pipe stem tube" (case 1), "cobble stone tube" and the "leopard skin tube" (4). Tubal calcification can take the form of linear streaks, which lie in the course of the fallopian tube or appear as faint or dense tiny nodules (as depict in case 4) (5). Calcification of the fallopian tubes or ovaries must be differentiated from calcified pelvic nodes, calcified uterine myomas, pelvic phleboliths and calcification in an ovarian dermoid (5).

Tubal occlusion is the most common HSG finding appeared in genital tuberculosis, which occurs most commonly in the region of isthmus and ampulla (1, 5). We noticed tubal occlusion in all cases (4, 19). Multiple constrictions along the course of the fallopian tube produce beaded appearance (Figure 2). Wide spectrum of occlusion and scars make rigidity in tubes and gives it a pipe stem appearance (Figure 5).

Healing produce densed tissue with scar around tubes which decrease tubes motility and makes them fixed so pritubal adhesion in HSG is revealed. Caseous ulceration of the mucosa of the tube make an irregular contour and diverticular outpouching which may surround the ampulla (tufted appearance) (Figure 6) or the isthmus (SIN view) (Figure 7) (5). The uterine changes in tuberculosis could be categorized as non-specific changes like evidence of endometritis, intrauterine adhesions, asymmetric uterine cavity and specific appearances such as collar-stud abscess, the tuberculosis T-shaped uterus (like in case3) and the pseudounicornuate uterus (4, 5, 15, 19). TB may confuse with other pathologies which may mimic non-specific features of uterine changes (4, 18, 19). 

Uterine changes vary from mild and normal view to irregular wall, deformity, asymmetry and obliteration in uterine cavity. TB’s scar cause triangular uterine cavity and make T-shape or septate appearance (Figure 8). Unilateral obliteration followed by unilateral scar in uterine cavity and revealed pseudo unicornuate appearance. Netter Syndrome is a severe degeneration and fibrosis with complete obstruction of uterine cavity. HSG shows glove’s finger appearance that only cervical canal and small portion of uterine cavity may be detected (Figure 9). 

Due to progressive endometrial lesion contrast medium may passed through lymphatic and venous systems (Figure 3B). Finally lymph node calcification, tubal and uterine cavity changes in HSG are so helpful in diagnosis. However, some of them are not specific. The diagnostic criteria established by Klein *et al* are very useful for TB diagnosis and described as follow (20):

1. There is calcified lymph node or irregular calcifications in adnexal area.
2. Obstruction of the fallopian tube in the zone of transition between the isthmus and ampulla.
3. Multiple constrictions along the course of the fallopian tube.
4. Endometrial adhesions and/or deformity or obliteration of the endometrial cavity in the absence of a history of curettage or abortion could be detected.

TB should always be considered in the differential diagnosis of a patient with an abdominal/ pelvic mass and ascites. Imaging findings can be misleading and diagnosis of abdominopelvic TB requires different clinical and laboratory evaluation (21). HSG is a good and simple imaging technique and can identify the lesions in more than 70% of cases (10).
